# Comprehensive Clinical Assessment of Patients With Acute Organophosphorus Poisoning in a Tertiary Care Hospital

**DOI:** 10.7759/cureus.69604

**Published:** 2024-09-17

**Authors:** Sharitha Kolli, Abhishek Burra, Naveenkumar Nallathambi, Reemu Bansal, Tanisha Rathi, Lakshya Choudhary, Mohit Agrawal, Tejaswee Lohakare, Gaurav Mittal

**Affiliations:** 1 Internal Medicine, Prathima Institute of Medical Sciences, Karimnagar, IND; 2 Emergency Medicine, Aadhya Hospital, Khammam, IND; 3 Internal Medicine, Madras Medical College, Chennai, IND; 4 Internal Medicine, Marengo Asia Hospitals, Faridabad, IND; 5 Internal Medicine, Mahatma Gandhi Institute of Medical Sciences, Wardha, IND; 6 Physical Medicine and Rehabilitation, S.S. Agrawal Institute of Physiotherapy and Medical Care Education, Navsari, IND; 7 Child Health Nursing, Srimati Radhikabai Meghe Memorial College of Nursing, Wardha, IND; 8 Research and Development, Student Network Organization, Mumbai, IND

**Keywords:** fatality rates, morbidity, op substances, patient characteristics, risk assessment, toxicity

## Abstract

Background

The use of organophosphorus (OP) compounds as pesticides is widespread, particularly in developing nations such as India. These substances are easily accessible and often linked to suicide poisoning incidents, especially among distressed farmers. In India, OP poisoning remains a significant cause of emergency hospital admissions. This study aims to analyze the clinical profile of patients admitted with OP poisoning, focusing on the various clinically significant characteristics and aspects associated with these cases.

Methods

Throughout the course of two years (from June 2022 to May 2024), five hospitals with critical care units participated in this prospective observational study. These hospitals referred patients to the Prathima Institute of Medical Sciences, which served as the central facility for the study. The study focused on individuals who arrived at the emergency room within 24 hours of acute exposure to organophosphate pesticides. A total of 150 patients meeting the inclusion criteria were enrolled. Data collection included a comprehensive clinical examination, detailed patient history, and monitoring of the response to intravenous atropine. The severity of the poisoning was evaluated using the Peradeniya Organophosphorus Poisoning (POP) scale, adapted for this study. Statistical analysis was performed using IBM SPSS Statistics for Windows, Version 23 (Released 2015; IBM Corp., Armonk, New York), with descriptive statistics used to summarize the categorical and continuous variables.

Results

The results of the study demonstrated a notable relationship between the arrival time at the health facility and mortality rates among patients with OP poisoning. Patients who arrived within three hours had a mortality rate of 13.2% (five out of 38 cases), while those arriving between three and six hours had a slightly higher rate of 14.3% (10 out of 70 cases). The mortality rate sharply increased to 28.6% (12 out of 42 cases) for patients who arrived after six hours. Among the OP compounds, monocrotophos showed the highest mortality rate at 80% (eight out of 10 cases), followed by dimethoate with 42.9% (12 out of 28 cases) and dichlorvos with 62.5% (five out of eight cases). The overall mortality rate in the study was 22.7% (34 out of 150 cases).

Conclusions

Poisoning with OP has primarily impacted young, productive men. Considerable mortality results from a disease condition that is more severe at presentation. It is advised to selectively outlaw the riskier substances that account for a larger percentage of fatalities.

## Introduction

Organophosphorus (OP) compounds are among the most widely used pesticides globally, especially in agricultural settings. In developing countries like India, where agriculture plays a pivotal role in the economy, these substances are readily available, leading to an increased risk of intentional poisoning, particularly among farmers facing high levels of distress. The easy accessibility and potent toxicity of OP compounds have made them a significant cause of suicide attempts, contributing to the alarming rate of poisoning cases. In India, OP poisoning remains one of the leading causes of emergency hospital admissions, posing a critical public health challenge [1]. The World Health Organization (WHO) estimates that approximately three million cases of pesticide poisoning occur annually worldwide, resulting in over 300,000 deaths [2].

The occurrence of OP poisoning is significantly more frequent in young adults of working age, with reported mortality rates ranging from 4% to 30% [3-5]. These figures underscore the severe impact of OP poisoning on public health, particularly in regions where agricultural practices are prevalent. Addressing the risk of OP poisoning requires a comprehensive strategy that includes effective public health responses, preventive measures, and timely medical interventions. The development of such responses is essential to reduce the burden of OP poisoning. Hospital management of these cases, including prompt and appropriate treatment, plays a crucial role in improving patient outcomes. Given the public health significance of OP poisoning, it is imperative to investigate and evaluate the diverse clinically significant characteristics and outcomes linked to these cases. The current study aims to provide a detailed clinical profile of OP poisoning patients admitted to a tertiary care center, with the goal of informing and enhancing the management strategies for such cases.

## Materials and methods

Study design and setting

Throughout the course of two years (from June 2022 to May 2024), five hospitals with critical care units participated in this prospective observational study. These hospitals referred patients to the Prathima Institute of Medical Sciences, which served as the central facility for the study. During the study period, all patients who had a history of acute OP poisoning and presented to the emergency room were included in the enrollment process. All eligible individuals who met the eligibility requirements throughout the specified study duration were incorporated into the research. Hence, no sample size calculation was performed, and no method for sampling was necessary. The study proceeded only after obtaining clearance from the ethics committee. All participants provided written, informed consent before being included in the research.

Selection Criteria

The study included patients with a history of acute OP poisoning within 24 hours of admission, presenting with muscarinic symptoms (e.g., miosis, excessive salivation, bronchorrhea, bradycardia), nicotinic symptoms (e.g., muscle fasciculations, weakness), and CNS symptoms (e.g., seizures, confusion, or coma). Patients who had ingested other toxic substances, such as alcohol or drugs, were excluded to prevent confounding effects on clinical presentation and outcomes. The sample size of 150 patients was determined based on the total number of patients who presented to the hospital with acute OP poisoning during the study period. Rather than pre-selecting a fixed sample size, all eligible patients who met the inclusion criteria and arrived within 24 hours of exposure were enrolled to capture a comprehensive range of clinical outcomes.

Data sources and variables

The emergency department (ED) was used to admit the participants. In the ED, skin decontamination procedures included gastric lavage, cleansing the skin and hair with soap and water, and removing contaminated clothing. OP poisoning was identified based on a detailed clinical examination, patient history, and the reaction to intravenous atropine. The severity of the poisoning was classified using the Peradeniya Organophosphorus Poisoning (POP) scale, which categorizes the severity into three grades: Grade I (mild), Grade II (moderate), and Grade III (severe). During the hospital stay, atropine and oximes were administered as needed, and patients were advised to follow up appropriately after discharge [6].

Statistical analysis

Microsoft Excel (Microsoft Corporation, Redmond, Washington) was used to enter the data into a master graphic, and IBM SPSS Statistics for Windows, Version 23 (Released 2015; IBM Corp., Armonk, New York) was used for analysis. Categorical and continuous variables were described using descriptive statistics, such as mean, percentage, and frequency analyses. Data from statistics were shown as mean ± standard deviation (SD).

## Results

Table 1 presents the demographic distribution of OP poisoning incidence among the study participants. The study included 150 participants, with a male predominance of 88 individuals (58.67%) and 62 females (41.33%). The age group 21-30 years was the most affected, accounting for 68 participants (45.33%), followed by those over 40 years old with 32 participants (21.34%). Participants under 20 years comprised 26 individuals (17.33%), and those aged 31-40 years included 24 participants (16%).

**Table 1:** Demographic distribution of organophosphorus (OP) poisoning incidence among study participants

Variables	Count	Percentage
Gender		
Male	88	58.67%
Female	62	41.33%
Age group		
Less than 20 years	26	17.33%
21-30 years	68	45.33%
31-40 years	24	16%
More than 40 years	32	21.34%

Table 2 outlines the distribution of 150 participants by their occupation, educational status, and region. Among the participants, farmers constituted the largest group, with 45 individuals (30.0%), followed by students (37 individuals, 24.67%) and laborers (30 individuals, 20.0%). In terms of educational status, the majority were illiterate (64 individuals, 42.67%), while those with high school education accounted for 38 participants (25.33%). Regarding the region, the majority of the participants (105 individuals, 70.0%) were from rural areas, with the remaining 45 participants (30.0%) from urban areas.

**Table 2:** Participant distribution by occupation, education level, and region

Variables	Count	Percentage
Occupation		
Farmer	45	30%
Student	37	24.67%
Laborer	30	20%
Housewife	23	15.33%
Unemployed	7	4.67%
Entrepreneur	4	2.67%
Employment (service)	4	2.67%
Educational level		
Uneducated	64	42.67%
Secondary education	38	25.33%
Elementary education	22	14.67%
Middle school	20	13.33%
College degree	6	4%
Area		
Rural	105	70%
Urban	45	30%

Table 3 details the mode and route of OP compound poisoning among 150 cases. The majority of the cases were suicidal, accounting for 145 instances (96.67%), with accidental exposure occurring in five cases (3.33%). There were no cases of homicidal exposure. Similarly, the primary route of administration was oral (145 cases, 96.67%), with skin exposure being the route in five cases (3.33%).

**Table 3:** Modes and routes of organophosphorus compound poisoning

Variables	Count	Percentage
Exposure method		
Intentional (suicidal)	145	96.67%
Unintentional (accidental)	5	3.33%
Intentional (homicidal)	0	0
Administration route		
Oral ingestion	145	96.67%
Dermal exposure	5	3.33%

Table 4 illustrates the relationship between the severity of poisoning, hospital stay, and mortality among the cases studied. The data reveals that Grade I poisoning, which accounted for 37 cases (24.67%), had an average hospital stay of three days, with five deaths occurring, representing 13.51% of these cases. Grade II poisoning, observed in 68 cases (45.33%), had a longer average hospital stay of five days, with 10 deaths, resulting in a mortality rate of 14.71%. The most severe cases, categorized as Grade III poisoning, were found in 45 patients (30%), who required an average of seven days in the hospital. This group had the highest mortality rate, with 19 deaths, accounting for 42.22% of the cases in this category. Overall, across all severity levels, the total number of cases was 150, with an average hospital stay of 5.13 days and a mortality rate of 22.67%, represented by 34 deaths.

**Table 4:** Relationship between poisoning severity, hospital stay, and mortality Grades I, II, and III represent the severity of organophosphorus poisoning as classified by the Peradeniya Organophosphorus Poisoning (POP) scale. Grade I indicates mild poisoning, Grade II represents moderate poisoning, and Grade III reflects severe poisoning.

Severity Level	Cases (n)	Cases (%)	Average Hospital Stay (Days)	Deaths (n)	Deaths (%)
Grade I	37	24.67%	3	5	13.51%
Grade II	68	45.33%	5	10	14.71%
Grade III	45	30%	7	19	42.22%
Total	150	100%	5.13	34	22.67%

Table 5 highlights the association between the arrival time at the health facility, the type of OP compound consumed, and patient mortality rates. The data show that patients who arrived at the health facility within three hours of poisoning had a mortality rate of 13.2%, with five deaths out of 38 cases. Patients who arrived between three and six hours had a slightly higher mortality rate of 14.3%, with 10 deaths out of 70 cases. The mortality rate significantly increased to 28.6% for those who arrived more than six hours after poisoning, with 12 deaths out of 42 cases. The chi-square test for arrival time shows a value of 4.59 with a p-value of 0.101, indicating that the association between arrival time and mortality is not statistically significant. When examining the specific OP compounds consumed, the highest mortality rate was observed in patients who ingested monocrotophos, with eight deaths out of 10 cases, resulting in an 80% mortality rate. Dimethoate was associated with a mortality rate of 42.9%, with 12 deaths out of 28 cases. Dichlorvos also had a high mortality rate of 62.5%, with five deaths out of eight cases. Chlorpyriphos had a mortality rate of 31.3%, with five deaths out of 16 cases. Other compounds, such as diazinon, metacid, and quinalphos, had lower mortality rates of 11.1%, 6.7%, and 33.3%, respectively. Notably, compounds such as paraoxan, malathion, dimecron, mevinphos, and fenthion were not associated with any deaths in the cases studied. The chi-square test for OP compounds shows a value of 39.35 with a p-value of <0.001, indicating a statistically significant association between the type of OP compound consumed and mortality. Overall, the total mortality rate across all cases was 22.7%, with 34 deaths among the 150 cases.

**Table 5:** Association of arrival time and type of organophosphorus compound with mortality

Death-Associated Factors	Cases (n)	Deaths (n)	Deaths (%)	Chi-Square Value	p-value
Arrival time at the health facility					
<3 hours	38	5	13.2%	1.60	0.448
3-6 hours	70	10	14.3%	2.44	0.295
>6 hours	42	12	28.6%	0.55	0.759
Total	150	27	18%	4.59	0.101
OP compound consumed					
Dimethoate	28	12	42.9%	5.46	0.020
Diazinon	18	2	11.1%	1.05	0.309
Chlorpyriphos	16	5	31.3%	1.00	0.317
Metacid	15	1	6.7%	5.60	0.018
Paraoxan	14	0	0	3.16	0.076
Malathion	13	0	0	2.94	0.086
Dimecron	12	0	0	2.58	0.108
Mevinphos	11	0	0	2.48	0.115
Monocrotophos	10	8	80%	15.62	<0.001
Dichlorvos	8	5	62.5%	5.52	0.019
Quinalphos	3	1	33.3%	0.15	0.699
Fenthion	2	0	0	0.45	0.504
Total	150	34	22.7%	39.35	<0.001

Table 6 summarizes the symptoms observed in patients with OP poisoning. The most common symptom was constricted pupils, observed in 112 patients, accounting for 74.7% of the cases. This was followed by increased secretions in 88 cases (58.7%) and muscle twitching in 60 cases (40%). Reduced consciousness was noted in 55 patients (36.7%), while excessive sweating was observed in 48 cases (32%). Other symptoms included loose stools in 46 patients (30.7%), stomach pain in 42 patients (28%), and elevated temperature in 44 patients (29.3%). Breathing difficulties were the least common, affecting 38 patients, which accounted for 25.3% of the cases.

**Table 6:** Symptoms observed in patients with organophosphorus poisoning

Symptom	Number of Cases	Percentage (%)
Constricted pupils	112	74.7%
Increased secretions	88	58.7%
Muscle twitching	60	40%
Reduced consciousness	55	36.7%
Excessive sweating	48	32%
Loose stools	46	30.7%
Stomach pain	42	28%
Elevated temperature	44	29.3%
Breathing difficulties	38	25.3%

## Discussion

The purpose of this study was to provide useful insight into a variety of clinically important characteristics of severe OP poisoning incidents presenting at the hospital. This allowed the cases to be examined for the most appropriate immediate/short-term and long-term therapeutic and preventative treatments. Data were evaluated, and 150 patients in total were enrolled. The current study discovered that young individuals (ages 21 to 30) and men were comparatively more impacted. The age group affection matched the findings of Guven et al. [7], who stated that the participants' mean age was 24.1 years. Similarly, Dassanayake et al. [8] reported that 91% of their cases included people under 30. The 54% skew is consistent with a male gender preference documented in the research by Sungur et al. [2]. In India, farming, which is primarily done in rural regions and includes a large number of illiterate or somewhat educated people, is the primary use of OP compounds. Therefore, it makes sense that individuals who are farmers, uneducated, or live in rural regions would be disproportionately affected. These results align with the findings of Otto et al. [9] in a study conducted in Germany and Gorea et al. [10] in research conducted in North India. It is not unexpected that the oral route of OP poisoning, which is suicidal in nature, accounts for nearly all instances, considering the chemicals' widespread use, easy accessibility, and free availability in India.

According to Sungur et al. [2] and Saadeh et al. [11], two-thirds of the cases were suicidally motivated, corresponding to 68% of the instances in this study. The reason for the discrepancy could be because the farmers are in hardship while having easy access to the dangerous OP chemicals. Using modified Dreisbach criteria, the degree of poisoning severity was assessed and graded. It was discovered that there was a clear correlation between the length of hospital stay and mortality, with the Grade III group reporting the greatest mean duration of hospital admission (seven days) and the highest mortality (19 deaths). This supports the conclusions made by Arup [12] in his West Bengal study. The current study's mean length of hospital stay was 5.13 days, aligning with previous comparable research [2,10-12]. The most common symptom, impacting 112 subjects (74.7%), was puffer constriction. Constriction of the pupils is the most prevalent symptom that is associated with acute OP poisoning, according to Thunga et al. [13]. However, the most frequent clinical symptoms that present themselves vary according to the study, with some claiming that they are related to the substance ingested. These include altered sensorium, diarrhea, profuse secretions, and stomach discomfort [14-16].

The current study found that the length of hospital stay and the mortality rate were negatively impacted by the time it took to get to the medical institution. This is consistent with earlier research that looked at the travel time to the medical institution and the underlying mechanisms of the disease progression in instances of OP poisoning [17]. In the current investigation, dimethoate was the most frequently occurring chemical responsible (28 cases, 42.9%) for poisoning. Dimethoate was shown to be the most often eaten OP chemical in Sri Lanka by Dassanayake et al. [8]. However, the research done by Sungur et al. [2] claimed that 51.1% of the cases were caused by dichlorvos. The choice of a particular compound may have been influenced by local enterprises manufacturing the compound or by the broad differences in expense and accessibility of the substance locally, depending on the crops cultivated in the area. Similar to this study, several earlier research of a similar nature have also revealed that monochrotophos is nearly always lethal. A frail primary healthcare system could be a contributing factor, in addition to a lack of public awareness, for the overall death rate of 34 (22.67%), which is somewhat higher than average.

Limitations of the study

Two notable drawbacks of the current study are the restricted sample size and the fact that the majority of the patients are from relatively rural areas. A larger investigation with a more diversified study group is recommended.

## Conclusions

There seems to be a greater effect of OP poisoning among young, economically active men. Substantial mortality results from increased illness severity at presentation and delayed reporting. It is advisable to selectively restrict the substances that pose a higher risk of mortality, in addition to improving the effectiveness and responsiveness of basic healthcare services in rural areas.
